# Loliolide in *Sargassum horneri* Alleviates Ultrafine Urban Particulate Matter (PM 0.1)-Induced Inflammation in Human RPE Cells

**DOI:** 10.3390/ijms25010162

**Published:** 2023-12-21

**Authors:** Eun Jeoung Lee, Sol Lee, Hyun-Jae Jang, Wonbeak Yoo

**Affiliations:** 1 AceBiome Inc., Seoul 06164, Republic of Korea; ejlee@acebiome.com (E.J.L.); slee@acebiome.com (S.L.); 2 R&D Center, AceBiome Inc., Daejeon 34013, Republic of Korea; 3 Natural Medicine Research Center, Korea Research Institute of Bioscience and Biotechnology, Cheonju-si 28116, Republic of Korea; water815@kribb.re.kr; 4 Natural Product Central Bank, Korea Research Institute of Bioscience and Biotechnology, Cheonju-si 28116, Republic of Korea; 5 Personalized Genomic Medicine Research Center, Korea Research Institute of Bioscience and Biotechnology, Daejeon 34141, Republic of Korea

**Keywords:** *Sargassum horneri*, ultrafine urban particulate matters, ARPE-19, anti-inflammation, MAPK/NF-ĸB

## Abstract

Owing to increasing air pollution due to industrial development, fine dust has been associated with threatening public health. In particular, ultrafine urban particulate matter (uf-UP, PM 0.1) can easily enter our bodies, causing inflammation-related diseases. Therefore, in the present study, we evaluated the effects of hydrothermal extracts of *Sargassum horneri* and its bioactive compound, loliolide, on uf-UP-induced inflammation as a potential treatment strategy for retinal disorders. Human retinal pigment epithelial cells (ARPE-19) stimulated with TNF-α or uf-UPs were treated with *S. horneri* extract and loliolide. *S. horneri* extracts exhibited anti-inflammatory effects on uf-UP-induced inflammation without cell toxicity through downregulating the mRNA expression of *MCP-1*, *IL-8*, *IL-6*, and *TNF-α*. UPLC-QTOF/MS analysis confirmed that the hydrothermal extract of *S. horneri* contained loliolide, which has anti-inflammatory effects. Loliolide effectively reduced the mRNA expression and production of proinflammatory chemokines (*IL-8*) and cytokines (*IL-1β* and *IL-6*) by downregulating the MAPK/NF-ĸB signaling pathway on TNF-α-stimulated inflammatory ARPE-19 cells. These effects were further confirmed in inflammatory ARPE-19 cells after stimulation with uf-UPs. Collectively, these results suggested the application of *S. horneri* as a functional ingredient for treating ocular disorders caused by particular matters.

## 1. Introduction

Environmental pollution has become increasingly severe worldwide, and many studies have reported that exposure to particulate matter (PM) harms human health. According to their aerodynamic diameters, PMs are divided into fine and ultrafine urban particulate matter (uf-UP), ranging from nanometers (nm) to microns (μm) [1]. Briefly, uf-UPs enter the body through the respiratory tract and are then translocated to all organs. Therefore, exposure to uf-UPs is closely correlated with respiratory diseases, such as asthma, chronic obstructive pulmonary disease (COPD), neurodegenerative diseases, as well as cardiovascular, metabolic, cancer, and emotional disorders [2,3,4]. In addition, PMs, including uf-UPs, cause eye diseases, such as allergic conjunctivitis, increased intraocular pressure, glaucoma, age-related macular degeneration (AMD), and dry eye syndrome [5,6,7,8].

*Sargassum horneri (S. horneri)* is a well-known brown alga commonly inhabiting the coast of Japan and Korea that is reported to exhibit various biological activities such as anti-inflammatory, anti-apoptotic and anti-allergic effects [9,10,11,12]. According to many studies, the main components of *S. horneri* mainly consist of polyphenols and carbohydrates [13,14]. *S. horneri* contains bioactive compounds, such as fucoidan and loliolide, with promising bioactivities, such as antioxidant and anti-inflammatory functions [15]. In particular, loliolide, the phenolic compound known as one of the active ingredients of *S. horneri*, is reported to have a variety of functions, such as cell protection, anti-cancer, anti-aging, and anti-apoptosis activities, including antioxidant and anti-inflammatory functions [16,17,18]. Furthermore, many studies have suggested that *S. horneri* is a potential inhibitor of PM-induced inflammatory responses, oxidative stress, and cell death [19,20]. We previously reported that fucoidan exhibits anti-inflammatory effects by suppressing the inflammatory response of TNF-α-stimulated ARPE-19 cells [14]. In particular, TNF-α, which is one of the key regulators of the PM-induced inflammatory response, is not only associated with dry eye syndrome and corneal epithelium inflammation but is also known to cause allergic immune responses that lead to the development of ocular allergy [8,21]. Nevertheless, the effects of uf-UPs on ocular disease remain relatively unknown compared with those of PMs.

In this study, we investigated the anti-inflammatory effects of *S. horneri* on uf-UP-stimulated ARPE-19 cells. We also evaluated whether loliolide reduces inflammation in a TNF-α-induced cellular model. We expect the present study will improve our knowledge of *S. horneri* and its functional compound, loliolide, which can relieve the uf-UP-induced inflammation of the retina.

## 2. Results

### 2.1. Cellular Changes in uf-UP-Exposed ARPE-19 Cells

**Figure 1:**
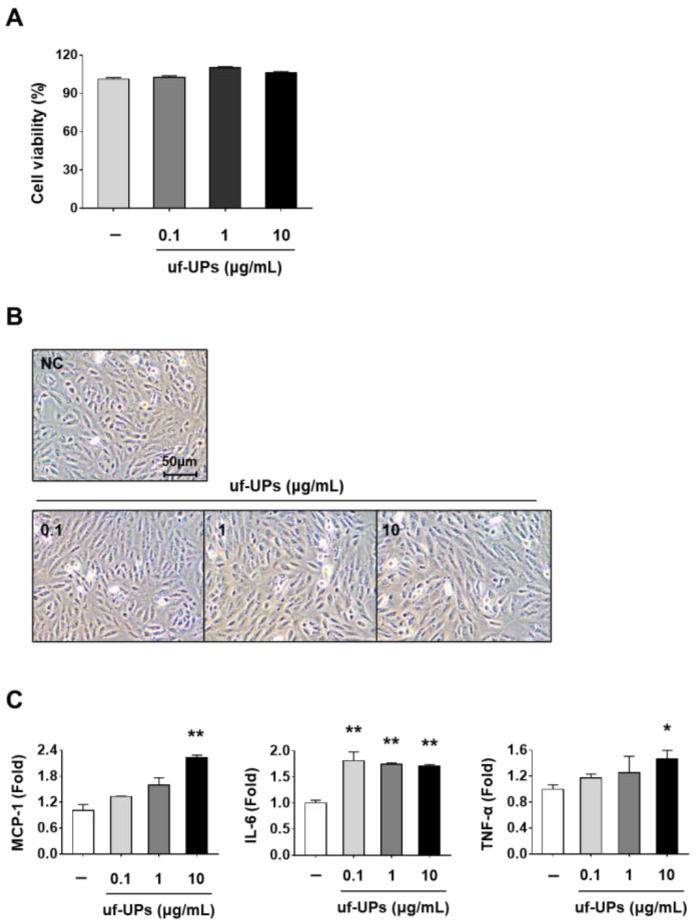
Cell viability and inflammation in uf-UP-treated ARPE-19 cells. ARPE-19 cells were treated with uf-UPs (0.1, 1, and 10 μg/mL). (**A**) Cell viability and (**B**) cell morphology 24 h after treatment. (**C**) Levels of mRNA expression of chemokines and cytokines in uf-UP-stimulated ARPE-19 cells. ARPE-19 cells were treated with uf-UPs (0.1, 1, and 10 μg/mL) for 1 h. Values represent the mean ± SD of three independent experiments. * *p* < 0.05 and ** *p* < 0.01 compared with the control.

We performed a cell viability assay to determine the toxicity of uf-UPs in APRE-19 cells. As shown in Figure 1A,B, no significant cytotoxicity or morphological changes were observed in cells exposed to concentrations of uf-UPs up to 10 μg/mL.

To determine whether uf-UP exposure induces an inflammatory response in ARPE-19 cells, we examined the levels of mRNA expression of inflammation-related genes using qRT-PCR. We observed that the mRNA levels of *MCP-1* and *TNF-α* were significantly increased in ARPE-19 cells exposed to 10 μg/mL uf-UPs, while the mRNA expression of *IL-6* was increased in cells treated with various concentrations of uf-UPs (Figure 1C). Therefore, we used 10 μg/mL uf-UPs for further experiments.

### 2.2. Effect of S. horneri on uf-UP-Induced Inflammation Response in ARPE-19 Cells

**Figure 2:**
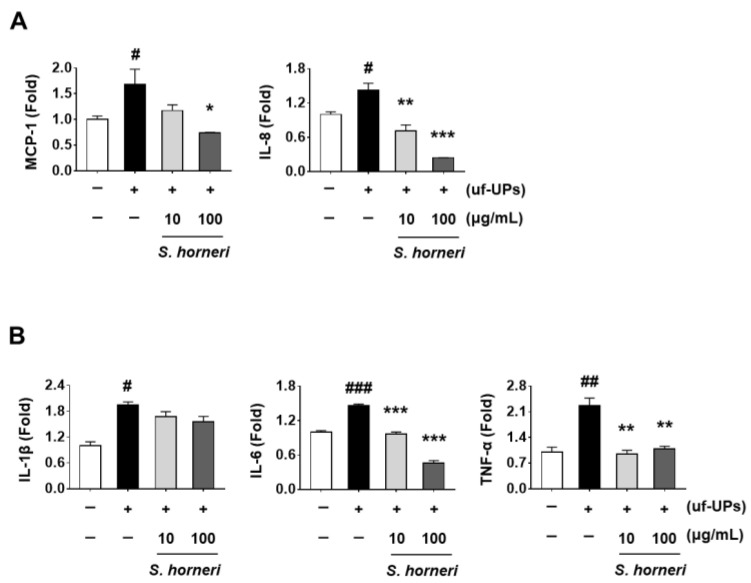
Effect of *S. horneri* on mRNA expression of inflammation-related genes in uf-UP-stimulated ARPE-19 cells. ARPE-19 cells were pretreated with *S. horneri* (10 and 100 μg/mL) for 1 h and then treated with 10 μg/mL uf-UPs for 1 h. Levels of mRNA expression of (**A**) chemokines *MCP-1* and *IL-8* and (**B**) cytokines *IL-1β*, *IL-6*, and TNF-α. Levels of mRNA expression were analyzed by RT-PCR. Values represent the mean ± SD of three independent experiments. # *p* < 0.05, ## *p* < 0.01, and ### *p* < 0.001 compared with the control. * *p* < 0.05, ** *p* < 0.01, and *** *p* < 0.001 compared with the uf-UP-treated control, respectively. +; treated TNF-α, −; non-treated TNF-α or loliolide.

To investigate whether *S. horneri* exerts anti-inflammatory effects on uf-UP-treated ARPE-19 cells, we analyzed the levels of mRNA expression of chemokines and cytokines. We found that exposure to uf-UPs significantly upregulated the mRNA levels of the *MCP-1* and *IL-8* chemokines, whereas pretreatment with *S. horneri* significantly downregulated their mRNA levels in a dose-dependent manner (Figure 2A). Although we detected that the mRNA level of *IL-1β* showed a decreasing trend after *S. horneri* pretreatment of cells under uf-UP-induced inflammatory conditions, it was not statistically significant. Of note, we determined that the mRNA expression of *IL-6* and *TNF-α* was significantly increased following exposure to uf-UPs. Conversely, we observed that *S. horneri* pretreatment significantly and dose-dependently reduced the upregulated expression of the above-mentioned genes (Figure 2B). These results suggested that *S. horneri* exerts anti-inflammatory effects on uf-UP-induced ARPE-19 cells.

### 2.3. UPLC-QTOF/MS Analysis

**Figure 3:**
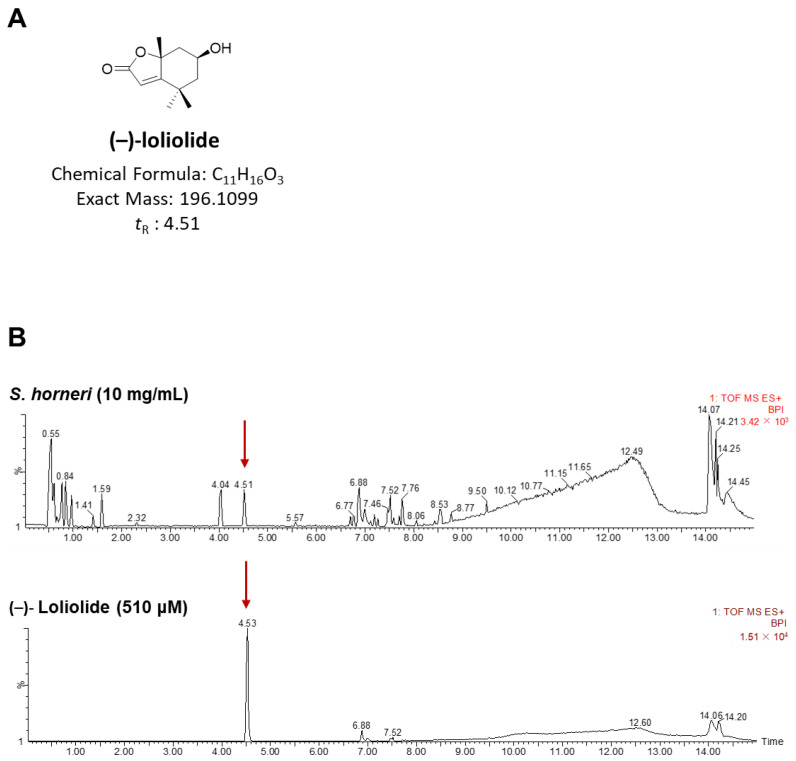
UPLC-QTOF/MS analysis. (**A**) Chemical structure of (–)-loliolide and (**B**) UPLC-QTOF/MS chromatogram of *S. horneri* aqueous extracts. Red arrow: BPI (Base Peak Intensity).

Based on the aforementioned results and a previous study [15], we considered loliolide to be the major component responsible for the anti-inflammatory effect of *S. horneri*. We, therefore, performed content analyses by chromatographically analyzing aqueous extracts of *S. horneri* using UPLC-QTOF/MS (Figure 3A). As shown in Figure 3B, the major peak observed at 4.5 min in the mass chromatogram of the *S. horneri* water extract corresponded to a protonated ion at *m*/*z* 197.1169 (Calcd for C_11_H_17_O_3_, 197.1178) in the positive mode. Based on optical rotation and ^1^H and ^13^C NMR spectral data, we identified this peak as corresponding to (–)-loliolide, a monoterpene lactone that was previously isolated from marine natural products such as brown algae and marine microalgae [22]. Based on the content analysis of *S. horneri*, we further investigated the in vitro anti-inflammatory properties of loliolide, which was presumed to be a major bioactive component of *S. horneri*.

### 2.4. Effect of Loliolide on Cell Viability in ARPE-19 Cells

**Figure 4:**
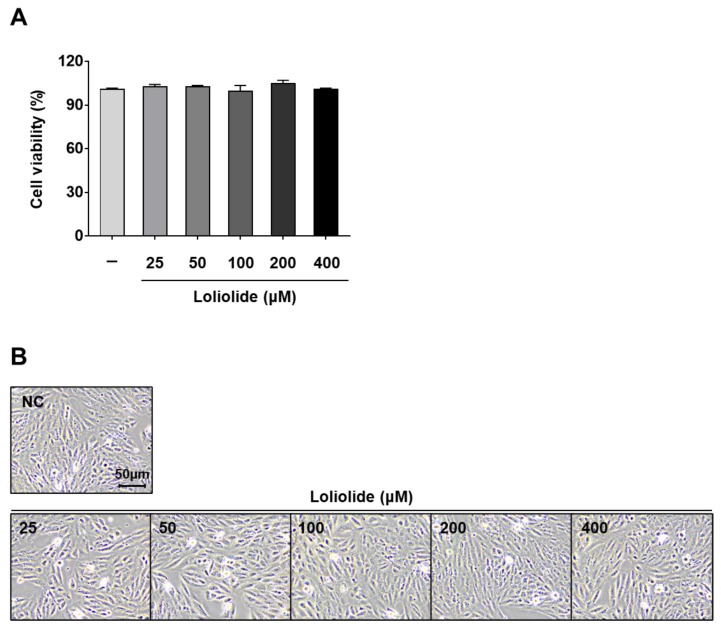
Cell viability and morphology in loliolide-treated ARPE-19 cells. ARPE-19 cells were treated with various concentrations of loliolide. (**A**) Cell viability and (**B**) cell morphology 24 h after treatment. Values represent the mean ± SD of three independent experiments.

To determine whether loliolide was cytotoxic to ARPE-19 cells, we measured cell viability using the MTT assay. We found that high concentrations of loliolide did not significantly affect the viability of ARPE-19 cells (Figure 4A) nor change their morphology (Figure 4B). Therefore, we used loliolide at a concentration below 400 μM to further evaluate its anti-inflammatory effect as a bioactive compound contained in *S. horneri*.

### 2.5. Effect of Loliolide on Inflammation in TNF-α-Stimulated ARPE-19 Cells

**Figure 5:**
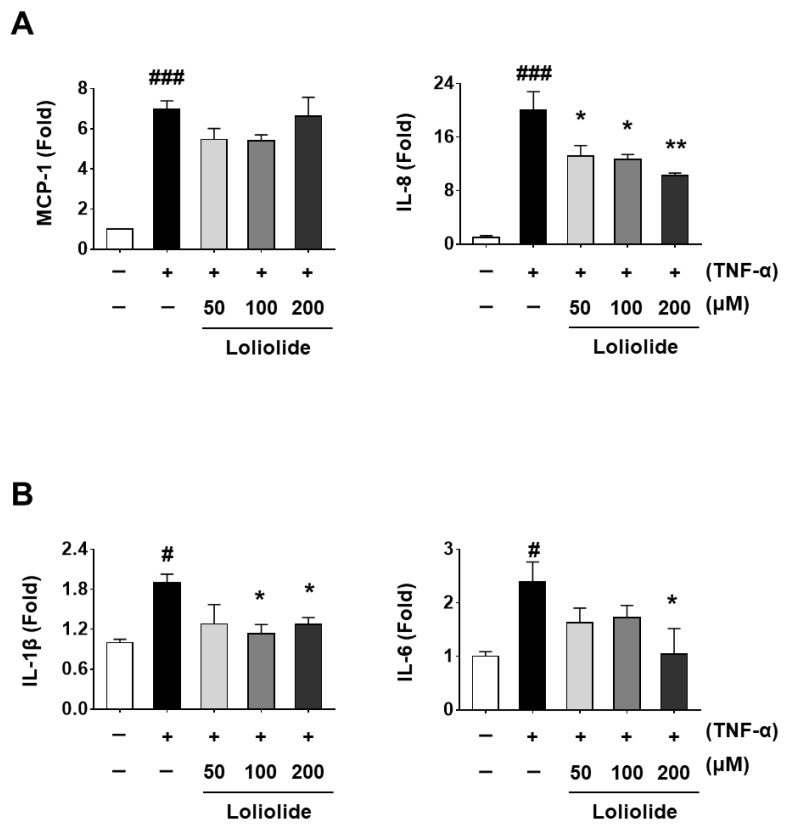
Effect of loliolide on mRNA expression of genes related to inflammation in TNF-α-treated ARPE-19 cells. ARPE-19 cells were pretreated with loliolide (50, 100, and 200 μg/mL) for 1 h and then treated with 50 ng/mL TNF-α for 4 h. Levels of mRNA expression of (**A**) chemokines *MCP-1* and *IL-8* and (**B**) cytokines *IL-1β* and *IL-6*. Levels of mRNA expression were analyzed by RT-PCR. Values represent the mean ± SD of three independent experiments. # *p* < 0.05 and ### *p* < 0.001 compared with the control. * *p* < 0.05 and ** *p* < 0.01 compared with the TNF-α-treated control, respectively. +; treated TNF-α, −; non-treated TNF-α or loliolide.

We next evaluated the anti-inflammatory effect of loliolide using a TNF-α-induced inflammatory cell model, which could activate the NF-ĸb signaling pathway and mimic aspects of PM-induced RPE inflammation. As shown in Figure 5A, the mRNA levels of chemokine *IL-8* were significantly decreased, whereas those of *MCP-1* was not changed after loliolide pretreatment of TNF-α-induced ARPE-19 cells. Regarding the mRNA expression of cytokine genes, we detected that the levels of *IL-1β* and *IL-6* were significantly decreased after loliolide pretreatment of TNF-α-induced ARPE-19 cells (Figure 5B).

**Figure 6:**
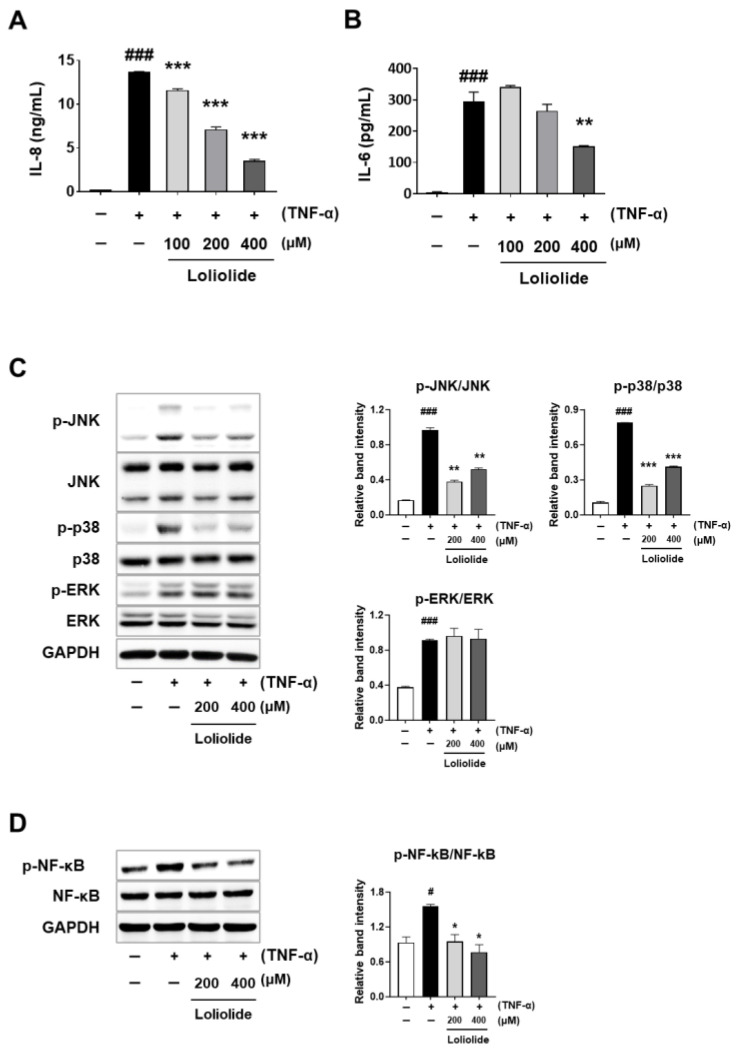
Effect of loliolide on cytokine production and MAPK/NF-κB signaling in TNF-α-treated ARPE-19 cells. ARPE-19 cells were pretreated with loliolide (100, 200, and 400 μM) for 1 h and then treated with 50 ng/mL TNF-α for 24 h. (**A**) Content of chemokine IL-8 and (**B**) cytokine IL-6. Phosphorylation of proteins in the (**C**) MAPK and (**D**) NF-κB signaling pathways. ARPE-19 cells were pretreated with loliolide (200 and 400 μM) for 1 h and then stimulated with 50 ng/mL TNF-α for 30 min. GAPDH was used as a loading control. Values represent the mean ± SD of three independent experiments. # *p* < 0.05, ### *p* < 0.001 compared with the control. * *p* < 0.05, ** *p* < 0.01 and *** *p* < 0.001 compared with the TNF-α-treated control, respectively. +; treated TNF-α, −; non-treated TNF-α or loliolide.

To further investigate the anti-inflammatory effects of loliolide, we measured the protein levels of chemokines and cytokines using ELISA. We accordingly found a significant upregulation in the production of IL-8 and IL-6 cytokines in TNF-α-induced ARPE-19 cells, which was conversely significantly and dose-dependently reduced by loliolide pretreatment (Figure 6A,B). To better understand the loliolide-induced anti-inflammatory response, we next performed Western blot analyses of proteins related to MAPK and NF-κB signaling, which are critical interconnected inflammatory pathways. We observed that loliolide pretreatment significantly attenuated the phosphorylation of JNK, p38, and NF-κB in TNF-α-treated ARPE-19 cells, whereas it did not affect ERK phosphorylation (Figure 6C,D). We then used the uf-UP-treated ARPE-19 model to extend and validate our previous findings to a TNF-α-induced ARPE-19 inflammation model. According to qRT-PCR results, the upregulated expression of MCP-1, IL-8, and IL-6 after treatment with uf-UPs was significantly decreased by loliolide pretreatment. (Supplementary Figure S4). Based on these results, we suggested that loliolide exhibits anti-inflammatory effects by inhibiting the activation of the NF-κB/MAPK signaling pathway in ARPE-19 cells under inflammatory conditions.

## 3. Discussion

The pathophysiological progression of retinal disease is closely linked to significant risk factors such as inflammation, which may affect vision and lead to vision loss. Increased ocular inflammation is associated with AMD, diabetic retinopathy (DR), and dry eye [23,24,25]. Because it is difficult to confirm or predict the progress of ocular disease, prevention by means of avoiding contact with toxic substances is important. Due to rapid industrial development, the use of carbon-containing fuels and traffic exhaust has increased, leading to increased PM emissions. Recent studies have reported that PMs can act as potent inducers of inflammatory responses in diverse tissues, including the eyes [26,27,28,29]. PMs generated reactive oxygen species (ROS) and activated stress kinase, such as JNK, whereas treatment of 2-isopropylmalic acid (2-IPMA) alleviated oxidative stress inflammatory response in RPE cells [30]. Sim et al. also showed that PMs enhance inflammation by upregulating the ROS-triggered apoptotic pathways via increases the ratio of Bax/Bcl-2, while sulforaphane treatment reversed these pro-apoptotic changes in ARPE-19 cells [31]. Despite evidence supporting the association between PM-mediated RPE inflammation and candidate for anti-inflammatory potential, it has not been thoroughly investigated, especially regarding the anti-inflammatory properties of natural marine ingredients. In the present study, we investigated the anti-inflammatory efficacy of *S. horneri* and loliolide, which is a bioactive compound of *S. horneri*, in uf-UP- and TNF-α-induced retinal cell inflammation.

PMs are classified according to their particle diameters, such as PM 10 (diameter less than 10 μm), PM 2.5 (diameter 2.5 to 10 μm), and PM 0.1 (also known as uf-UPs, diameter less than 0.1 μm), and are considered harmful factors affecting health as they can induce various pathologies, including cardiovascular toxicity, pulmonary diseases, hematological aberration, cutaneous inflammation, and metabolic syndrome [32,33,34,35,36]. Although uf-UPs are less well known than PM 2.5 and PM 10, recent studies have shown that uf-UPs pose a more serious danger to human health because they can be translocated into the bloodstream, spread to other organs, and have thus been positively associated with COPD, cardiovascular diseases, congestive heart failure, preterm birth, asthma, and acute myocardial infarction [37]. Therefore, effective prevention and treatment are required to reduce the health risks of exposure to uf-UPs.

Marine brown algae are an important food source and functional ingredients that are rich in bioactive compounds such as phlorotannins, fucoxanthin, alginic acid, fucoidans, and laminarin [38,39]. *S. horneri* extracts and its bioactive compounds, including loliolide, have demonstrated beneficial physiological activities such as antioxidant, antisenescence, and anti-inflammatory effects against external stress [40,41,42]. Despite the identification of various beneficial activities of loliolide, no studies have explored its effect on ocular inflammatory diseases, especially uf-UP-induced disorders.

The retinal pigment epithelium (RPE) eliminates external agents through its phagocytic function, while also transporting large amounts of nutrients to the eye [43,44]. In addition, the RPE regulates the activity of immune cells and maintains ocular immune privilege [45]. Owing to these interactions and internal or external stimuli, the RPE is relatively sensitive to stress, especially inflammation. Therefore, the inhibition of inflammatory responses that are upregulated by uf-UPs, one of the various external stimuli, is necessary to reduce the complications associated with long-term chronic inflammation, including ocular disease. In the current study, we found that uf-UPs stimulated inflammation in ARPE-19 cells by enhancing the mRNA expression of proinflammatory chemokines and cytokines, whereas pretreatment with *S. horneri* extract significantly attenuated the uf-UP-induced inflammation. In addition, mRNA expression analysis showed that the production of *MCP-1*, *IL-8*, *IL-6*, and *TNF-α* was reduced after pretreatment of uf-UPs-treated cells with *S. horneri* extracts. Based on previous studies and content analysis using UPLC-QTOF/MS, we confirmed that *S. horneri* contains loliolide (Figure 3). Therefore, we investigated whether loliolide improves the inflammatory response in ARPE-19 cells. Our results indicated that loliolide treatment decreased the mRNA expression of *IL-6* and *IL-8* in inflammatory ARPE-19 cells induced by uf-UPs and TNF-α treatment, whereas the effect on *MCP1* and *IL-1β* was only partial (Figure 5 and Supplementary Figure S4). Additionally, in our previous study, we confirmed that fucoidan has an anti-inflammatory effect against inflammation induced by TNF-α in ARPE-19 cells. Compared to these results, loliolide reduced chemokine secretion more than fucoidan [14].

## 4. Materials and Methods

### 4.1. Chemicals and Reagents

All chemicals and reagents were obtained from Sigma-Aldrich (St. Louis, MO, USA) unless stated otherwise. Thiazolyl blue tetrazolium bromide (MTT) powder was purchased from Tokyo Chemical Industry Co., Ltd. (Tokyo, Japan). TNF-α was purchased from PeproTech (Rocky Hill, NJ, USA). Loliolide was purchased from ChemFaces (Wuhan, China). The TRIzol reagent and bicinchoninic acid (BCA) protein assay kits were purchased from Thermo Fisher Scientific (Waltham, MA, USA). The MAPK Family Antibody Sampler Kit (cat. no. 9926) and phospho-MAPK Family Antibody Sampler Kit (cat. no. 9910) were purchased from Cell Signaling Technology (Danvers, MA, USA).

### 4.2. Preparation of S. horneri Powder

*S. horneri* was collected from the South Sea of Korea, and *S. horneri* powder preparation was performed as previously described [14]. Briefly, water extraction was performed at 100 °C for 3 h. The extract was further filtered (50 μm) and concentrated using a rotary vacuum evaporator (50 ± 10 °C). After concentration, freeze-drying was performed for approximately 72 h until the moisture content was less than 5%. The obtained powder was analyzed in a UPLC-QTOF/MS instrument (Waters, Milford, MA, USA) and used for further experiments.

### 4.3. Preparation of uf-UPs

uf-UPs were prepared using a previously reported method [46] with slight modifications. Briefly, 2 g of UPs (NIST1648A, Sigma, St. Louis, MO, USA) were added to 10 mL phosphate-buffered saline (PBS), vortexed for 1 min, sonicated for 45 min using an ultrasonic bath (Power Sonic 510, Hwashin Technology, Seoul, Republic of Korea), and filtered through a 0.22 μm syringe filter (Sartorius, Goettingen, Germany). These UPs were collected from St. Louis, MO, USA, and all constituents are described in the Certificate of Analysis (https://tsapps.nist.gov/srmext/certificates/1648a.pdf, accessed on 20 February 2023.

### 4.4. Cell Culture

The human RPE cell line ARPE-19 was grown in a 1:1 mixture of Dulbecco’s modified Eagle’s medium/Ham’s F-12 Nutrient Mixture (Welgene, Daegu, Republic of Korea) supplemented with 10% fetal bovine serum (HyClone™, Logan, UT, USA) and antibiotic/antimycotic solution (HyClone™) at 37 °C and in an atmosphere containing 5% CO_2_.

### 4.5. Cell Viability Assay

Cell viability was measured using the MTT assay. ARPE-19 cells were seeded into 12-well plates at a density of 1 × 10^5^ cells/well and incubated for 24 h. After incubation, uf-UPs (0.1, 1, and 10 μg/mL) or loliolide (25, 50, 100, 200, and 400 μM) was added at the indicated concentrations, and cells were further incubated for 24 h at 37 °C in humidified air with 5% CO_2_. The concentration of *S. horneri* was determined based on previous studies [14]. A stock solution of MTT (5 mg/mL in PBS) was added to each well at a final concentration of 0.5 mg/mL according to the manufacturer’s protocol. After incubation for 2 h at 37 °C, formazan was solubilized in dimethyl sulfoxide (DMSO). Cell viability was measured at 590 nm using a Multiskan™ SkyHigh microplate reader (Thermo Fisher Scientific). The percentage of treated cells exhibiting cytotoxicity was determined relative to that of the control group.

### 4.6. Instruments and Analytical Conditions

The aqueous extract of *S. horneri* was chromatographed using a C_18_ column (Waters BEH C_18_, 1.7 μm, 2.1 mm × 100 mm) on an ultraperformance liquid chromatography system (UPLC, ACQUITY, Waters) equipped with a Micromass QTOF Premier mass spectrometer (Waters). A solvent system consisting of 0.1% formic acid in water (A) and 0.1% formic acid in acetonitrile (B) was applied at a flow rate of 0.4 mL/min with the gradient method being as follows: 0.0–1.0 min, 10% B; 1.0–4.0 min, 20% B; 4.0–6.0 min, 40% B; 6.0–8.0 min, 85% B; 8.0–12.0 min, 100% B; 12.0–13.4 min, 100% B; 13.4–13.5 min, 10% B, 13.5–15.0 min, 10% B. The temperature of the column oven was kept at 35 °C, and the injection volume was 2 μL. The parameters applied for mass spectrometric analysis were the same as those used in the analysis method described by Jang et al. [47]. NMR spectroscopic data were recorded using a JEOL ECZ500R spectrometer (JEOL, Tokyo, Japan). Optical rotation was measured using a P-2000 polarimeter (JASCO, Tokyo, Japan). Reference chemical (–)-loliolide (CAS No. 5989-02-6) was supplied by the Korea Plant Extract Bank of the Korea Research Institute of Bioscience and Biotechnology (KRIBB, Daejeon, Republic of Korea). The NMR and MS spectroscopic data for (–)-loliolide are provided in the Supplementary data (Figures S1–S3).

### 4.7. RNA Preparation and Quantitative Real-Time PCR

Total RNA was extracted using the TRIzol^®^ reagent (Thermo Fisher Scientific), according to the manufacturer’s protocol. cDNA was synthesized using a RevertAid RT Reverse Transcription Kit (Thermo Fisher Scientific). Quantitative real-time PCR (real-time qPCR) was performed using the 2× GreenStar™ qPCR Master Mix (Bioneer, Daejeon, Korea), according to the manufacturer’s instructions. A total of 18S rRNA and GAPDH were used as reference genes for the normalization of all samples, and the levels of gene expression were calculated using a previously reported method [48]. All primers used are listed in Table S1.

### 4.8. Measurement of Cytokine and Chemokine Production

The amount of secreted cytokines in cell culture supernatants was determined using enzyme-linked immunosorbent assays (ELISA). The content of IL-6 (cat. no. ab178013) and IL-8 (cat. no. ab214030) in the supernatant was measured using relevant ELISA kits according to the manufacturer’s instructions (Abcam, Cambridge, UK).

### 4.9. Western Blot Analysis

To extract total protein, ARPE-19 cells were washed with cold PBS and lysed using a radioimmunoprecipitation assay (RIPA) buffer containing a protease and phosphatase inhibitor cocktail. The concentration of extracted proteins was measured using a BCA protein assay kit (Thermo Fisher Scientific) according to the manufacturer’s protocol. Samples of extracted total protein (20 μg/mL) were separated on 8% sodium dodecyl sulfate-polyacrylamide gel electrophoresis (SDS-PAGE) gels and transferred to polyvinylidene difluoride (PVDF) membranes. Membranes were blocked with 3% BSA or 5% skim milk for 1 h at room temperature. Membranes were then incubated with primary antibodies at 4 °C overnight. Primary antibodies included antibodies against p-JNK, JNK, p-p38, p-38, p-ERK, ERK, p-NF-κB, and NF-κB. After incubation, membranes were incubated with horseradish peroxidase (HRP)-conjugated secondary antibodies for 1 h at 25 °C. Protein bands were detected using SuperSignal™ West Pico PLUS Chemiluminescent Substrate (Thermo Fisher Scientific) and photographed under an iBright™ CL750 Imaging System (Thermo Fisher Scientific). GAPDH was used as the protein-loading control.

### 4.10. Statistical Analysis

All experiments were performed independently at least three times, and data were analyzed using GraphPad Prism 7.0 (San Diego, CA, USA). Data were expressed as the mean ± standard error of the mean. Statistical analyses were performed using Student’s *t*-test (for two-group comparisons) or one-way analysis of variance (for multiple-group comparisons). For all comparisons, a *p*-value < 0.05 was considered statistically significant.

## 5. Conclusions

In summary, this study suggested that *S. horneri* extract reduces the levels of proinflammatory markers in uf-UP-stimulated ARPE-19 cells. In a TNF-α-induced inflammation model, *S. horneri* extracts significantly alleviated proinflammatory phenotypes as indicated by the levels of mRNA expression and production of chemokines and cytokines via downregulating the MAPK/NF-ĸB signaling pathway. Likewise, loliolide treatment showed significant anti-inflammatory effects on uf-UP- and TNF-α-stimulated inflammation in ARPE-19 cells. Therefore, the present study demonstrated that *S. horneri* extract can be a good functional ingredient for alleviating retinal inflammatory diseases caused by external stimuli.

## Data Availability

Data are contained within the article and Supplementary Materials.

## Supplementary Materials

The following supporting information can be downloaded at: https://www.mdpi.com/article/10.3390/ijms25010162/s1.
