# Supplementary material for: Loliolide in Sargassum horneri Alleviates Ultrafine Urban Particulate Matter (PM 0.1)-Induced Inflammation in Human RPE Cells
Source: Int J Mol Sci. 2023 Dec 21;25(1):162. doi: 10.3390/ijms25010162 (PMC10779059; doi:10.3390/ijms25010162)
Supplement: Supplementary file 1 [file ijms-25-00162-s001.zip › ijms-2738693-supplementary/Supplementary Table.pdf]

Table 1. Sequences of the primers for RT-PCR

Primer	Sequence
Human GAPDH	F: 5’-CCACTCCTCCACCTTTGAC-3’
	R: 5’-ACCCTGTTGCTGTAGCCA-3’
Human 18s rRNA	F: 5’-ATCACCATTATGCAGAATCCACG-3’
	F: 5’-GACCTGGCTGTATTTTCCATCC-3’
Human MCP-1	F: 5’-CCCCAGTCACCTGCTGTTAT-3’
	R: 5’-GCTTCTTTGGGACACTTGCT-3’
Human IL-8	F: 5’-TTTTGCCAAGGAGTGCTAAA-3’
	R: 5’-CTCTGCACCCAGTTTTCCTT-3’
Human IL-1β	F: 5’-GGGACAGGATATGGAGCAACA-3’
	R: 5’-TTTCAACACGCAGGACAGGTA-3’
Human IL-6	F: 5’-ACTTTCACTTAAGACCCAGGGA-3’
	R: 5’-AGTGTTGAGATGATGCTTTGACA-3’
Human TNF-α	F: 5’-TGGCGTGGAGCTGAGAGATAA-3’
	R: 5’-TTGATGGCAGAGAGGAGGTTGA-3’
