# Supplementary material for: Loliolide in Sargassum horneri Alleviates Ultrafine Urban Particulate Matter (PM 0.1)-Induced Inflammation in Human RPE Cells
Source: Int J Mol Sci. 2023 Dec 21;25(1):162. doi: 10.3390/ijms25010162 (PMC10779059; doi:10.3390/ijms25010162)
Supplement: Supplementary file 1 [file ijms-25-00162-s001.zip › ijms-2738693-supplementary/Supplementary figures.pdf]

List of Supplementary data

Figure S1. HR-ESI-QTOF/MS mass spectrum of (–)-loliolide 3
Figure S2. ^{1}H NMR (500 MHz) spectrum of (–)-loliolide 4
Figure S3. ^{13}C NMR (125 MHz) spectrum of (–)-loliolide 5
Figure S4. Effect of loliolide on mRNA expression levels of inflammation marker in uf-UPs-treated ARPE-19 cells. 6

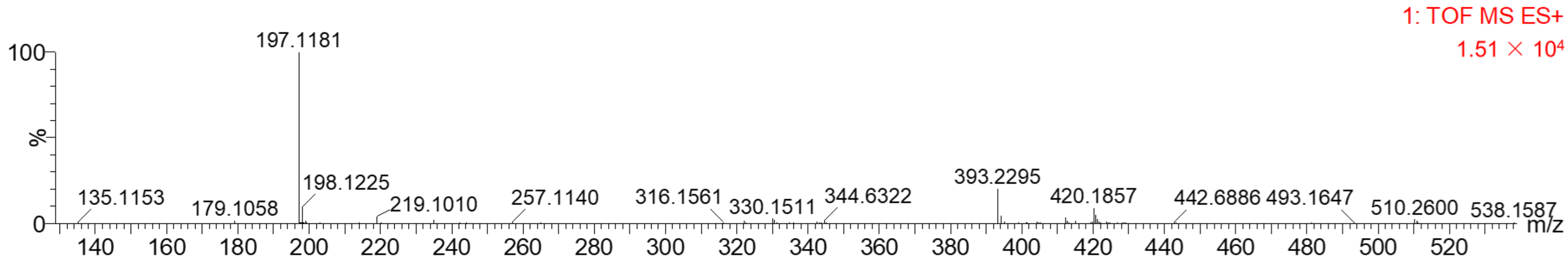

Mass	Calc. Mass	mDa	PPM	DBE	Formula	i-FIT	i-FIT Norm	Fit Conf %	C	H	N	O
197.1181	197.1178	0.3	1.5	3.5	C11 H17 O3	147.3	2.569	7.66	11	17		3

Figure S1. HR-ESI-QTOF/MS mass spectrum of (–)-loliolide.

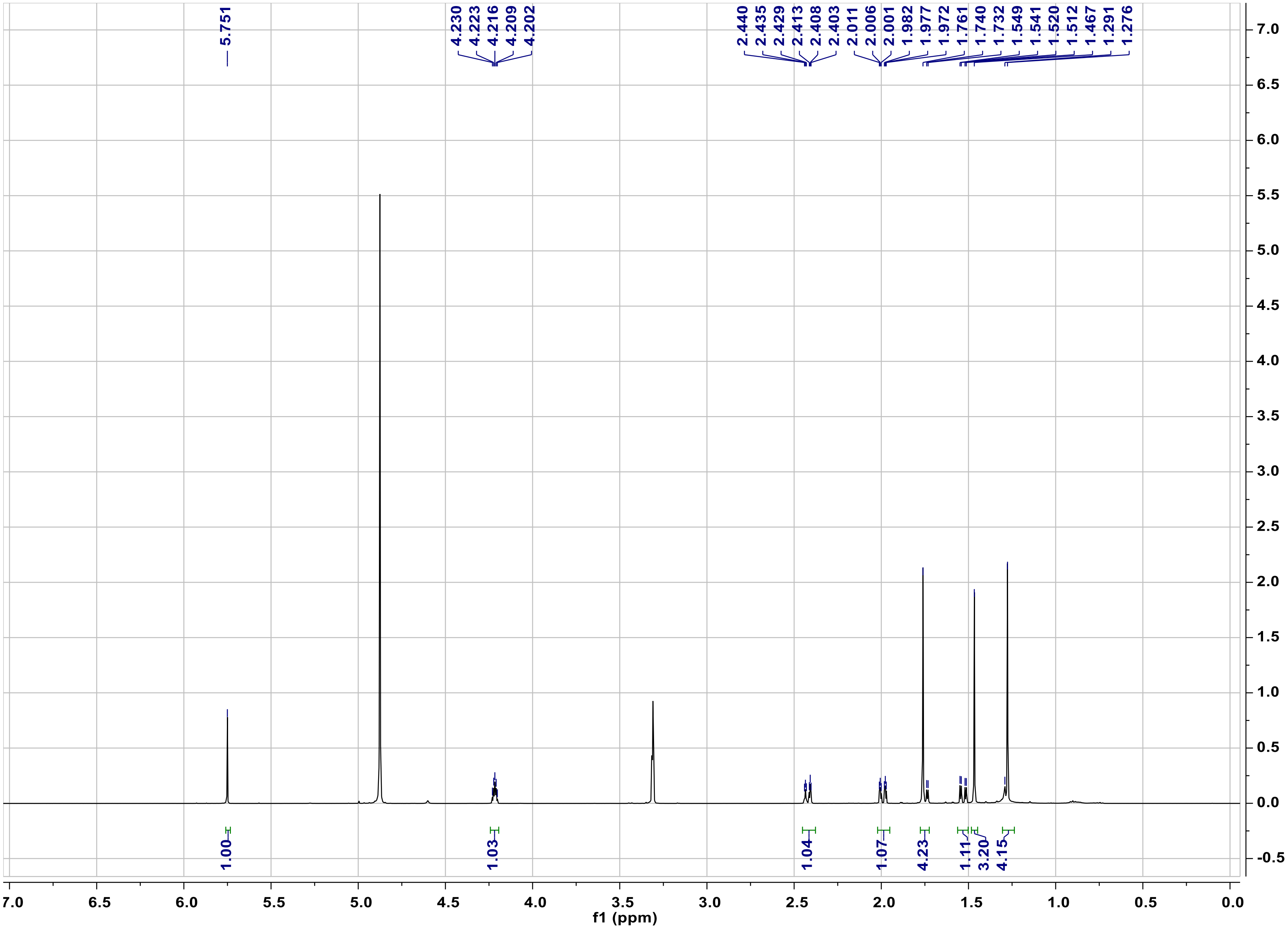

Figure S2. ^{1}H NMR (500 MHz) spectrum of (–)-loliolide.

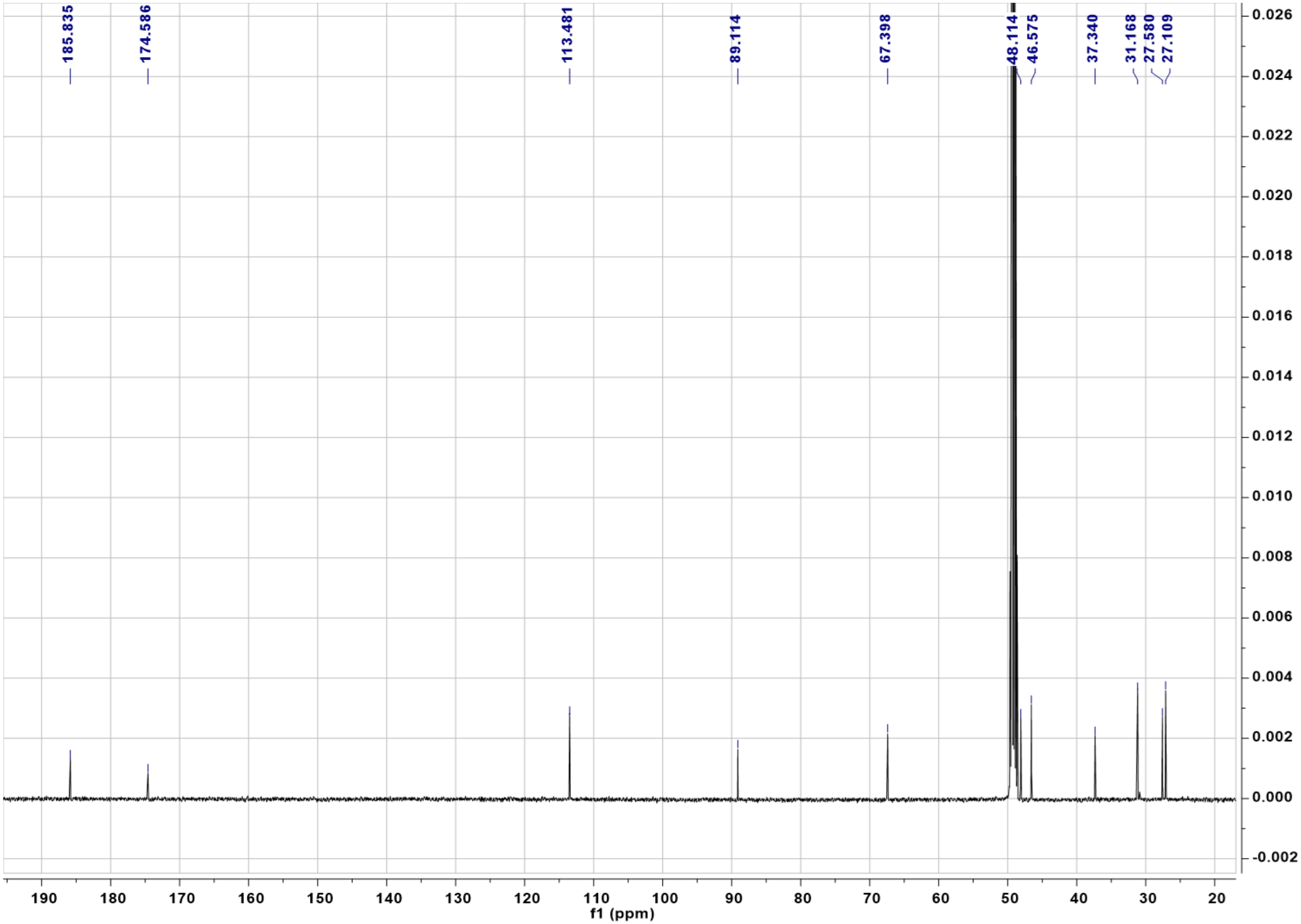

Figure S3. ^{13}C NMR (125 MHz) spectrum of (–)-loliolide.

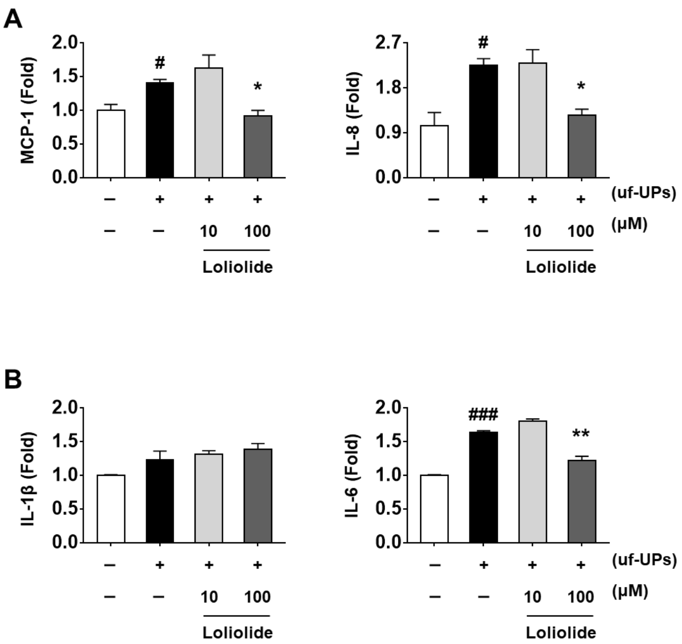

Figure S4. Effect of loliolide on mRNA expression levels of inflammation marker in uf-UPs-treated ARPE-19 cells. ARPE-19 cells were pre-treated with loliolide (10, 100 μM) for 1 h and then treated with 10 μg/mL uf-UPs for 1 h. mRNA expression levels of (A) chemokine *MCP-1* and *IL-8* and (B) cytokine *IL-1β* and *IL-6*. The values represent the mean ± SD of three independent experiments. # $p < 0.05$ and ### $p < 0.001$ compared with the control. * $p < 0.05$ and ** $p < 0.01$ compared with the uf-UPs-treated control, respectively.
